# A Mouse Model to Test Novel Therapeutics for Parkinson’s Disease: an Update on the Thy1-aSyn (“line 61”) Mice

**DOI:** 10.1007/s13311-022-01338-0

**Published:** 2023-01-30

**Authors:** Franziska Richter, Milos Stanojlovic, Christopher Käufer, Birthe Gericke, Malte Feja

**Affiliations:** 1 Department of Pharmacology, Toxicology and Pharmacy, University of Veterinary Medicine Hannover, Foundation, Bünteweg 17, 30559 Hannover, Germany; 2 Center for Systems Neuroscience Hannover, Hannover, Germany

**Keywords:** Parkinson’s disease, Alpha-synuclein, Progressive, Neuroprotection, Therapy

## Abstract

Development of neuroprotective therapeutics for Parkinson’s disease (PD) is facing a lack of translation from pre-clinical to clinical trials. One strategy for improvement is to increase predictive validity of pre-clinical studies by using extensively characterized animal models with a comprehensive set of validated pharmacodynamic readouts. Mice over-expressing full-length, human, wild-type alpha-synuclein under the Thy-1 promoter (Thy1-aSyn line 61) reproduce key features of sporadic PD, such as progressive loss of striatal dopamine, alpha-synuclein pathology, deficits in motor and non-motor functions, and elevation of inflammatory markers. Extensive work with this model by multiple laboratories over the past decade further increased confidence in its robustness and validity, especially for analyzing pathomechanisms of alpha-synuclein pathology and down-stream pathways, and for pre-clinical drug testing. Interestingly, while postnatal transgene expression is widespread in central and peripheral neurons, the extent and progression of down-stream pathology differs between brain regions, thereby replicating the characteristic selective vulnerability of neurodegenerative diseases. In-depth characterization of these readouts in conjunction with behavioral deficits has led to more informative endpoints for pre-clinical trials. Each drug tested in Thy1-aSyn line 61 enhances knowledge on how molecular targets, pathology, and functional behavioral readouts are interconnected, thereby further optimizing the platform towards predictive validity for clinical trials. Here, we present the current state of the art using Thy1-aSyn line 61 for drug target discovery, validation, and pre-clinical testing.

**Supplementary Information:**

The online version contains supplementary material available at 10.1007/s13311-022-01338-0.

## Challenges in Drug Development for Parkinson’s Disease and Implications for Animal Models Used in Pre-Clinical Trials

### Current State of Drug Therapy for Parkinson’s Disease

While current therapy effectively alleviates many symptoms caused by dopamine loss due to degeneration of dopaminergic neurons in the substantia nigra pars compacta, there is still no treatment to halt or modify the progression of underlying disease mechanisms in Parkinson’s disease (PD) or other synucleinopathies [1–3]. Importantly, non-motor symptoms such as cognitive dysfunction, anxiety, signs of depression, or constipation severely reduce quality of life for patients and caregivers, but all currently available treatments focus on symptomatic relief as opposed to halting underlying neurodegeneration [4, 5]. If there is no disease-modifying therapy discovered and applied prior to diagnosis, the aging world population is estimated to have 12.9 million people affected with PD by 2040 [6]. Models of PD used to uncover novel drug targets and to test therapeutics should encompass aspects of the disease complexity in etiology, pathology, and functional readouts.

It is now apparent that PD is not only a disease of the dopamine system but also includes many neuronal subtypes, alterations in diverse neuronal circuitries and considerable functional decline of the peripheral nervous system [7]. Hence, PD is a multi-system and multi-faceted syndrome manifesting and progressing uniquely for each patient. The cause (environment and/or genetics) and the site (central nervous system and/or periphery) from which neurodegenerative processes are initiated are likely as multi-faceted as the symptoms and the disease course [8, 9].

### Strategies to Improve Success in Clinical Trials with Disease-Modifying Therapeutics

**Fig. 1:**
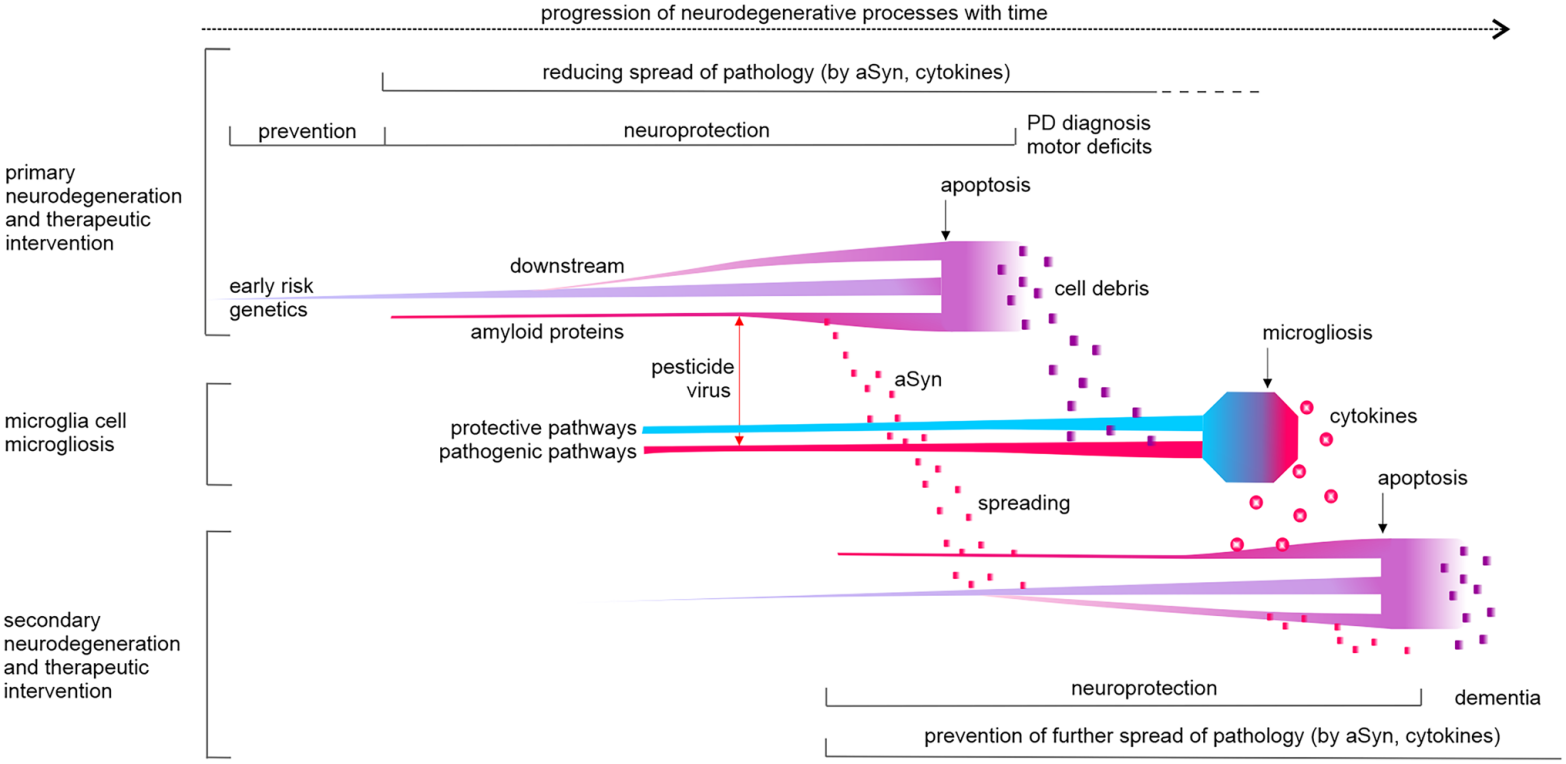
Exemplified progression of neurodegenerative processes and therapeutic interventions. For a specific neuron/neuronal population, there are early processes that predispose to neurodegeneration, which induce several downstream secondary processes that with time progress independently from the primary cause (e.g., GBA mutation to lysosomal dysfunction to proteinopathy to mitochondrial damage). Together with additional processes that initiate by a toxic event (pesticides, virus [17]), they create a burden that ultimately cumulates in cell death (apoptosis). Alpha-synuclein (aSyn) pathology may spread to other cells and induce degenerative processes. Cell debris from dying neurons initiate microgliosis and release of cytotoxic cytokines. At time of diagnosis, the majority of dopamine neurons is degenerated and the remaining neurons degenerate in a few years [18]. Neuroprotective therapy would have to substantially halt several neurodegenerative processes and probably reduce microgliosis for overt benefits on dopamine related endpoints. Solutions to enhance neuroprotective benefits on motor symptoms could be (i) starting prior to overt dopamine neuron loss, if early diagnosis becomes more reliable, or (ii) target several degenerative processes and the spreading to less affected neurons, which would require drug combinations. Alternatively, adapt clinical readouts away from classical motor symptoms to more sensitive pharmacodynamics biomarkers, if they become available, including non-motor symptoms projected to develop in stratified patient groups (e.g., cognitive decline in GBA mutation carriers). To address and foresee ongoing efforts and successes to overcome these challenges, pre-clinical studies need to cover dopamine related endpoints in combination with a comprehensive set of pharmacodynamic readouts to be informative for current and future clinical trial designs

Disease-modifying therapeutics target the disease course by preventing or halting neurodegenerative processes [10, 11] (Fig. 1). However, there is an ongoing gap to the clinics, where promising therapeutic candidates fail to reach primary endpoints [3]. Optimism is still warranted as there are a number of phase 3 trials in the planning stages and 13 disease-modifying therapeutics in phase 2 trials due to complete by the end of 2022 [12]. As explained in Fig. 1, intervention may need to target multiple facets of dopamine neuron degeneration in order to achieve therapeutic efficacy [13, 14]. Rigorous criteria to define early disease stages, risk and potential stratification criteria, as well as suitable quantifiable endpoints and non-clinical progression biomarkers, are not yet available for the individual case [1]. In recently diagnosed PD patients, more sensitive primary endpoints, such as fine motor control, improved neuroimaging (as biomarker) or selected non-motor symptoms are required; however, there are currently no clearly validated (effect size needs to be defined) progressive symptoms or biomarkers to this purpose. An enormous effort is being made to overcome these hurdles, and there is considerable progress in recent years [15, 16]. Thus, with these challenges and prospects in mind, current and future pre-clinical studies in animal models need to be optimized to provide best possible information on the prospect of a compound to be efficacious in clinics for a targeted endpoint.

### Validity Criteria for Animal Models in Drug Development

**Fig. 2:**
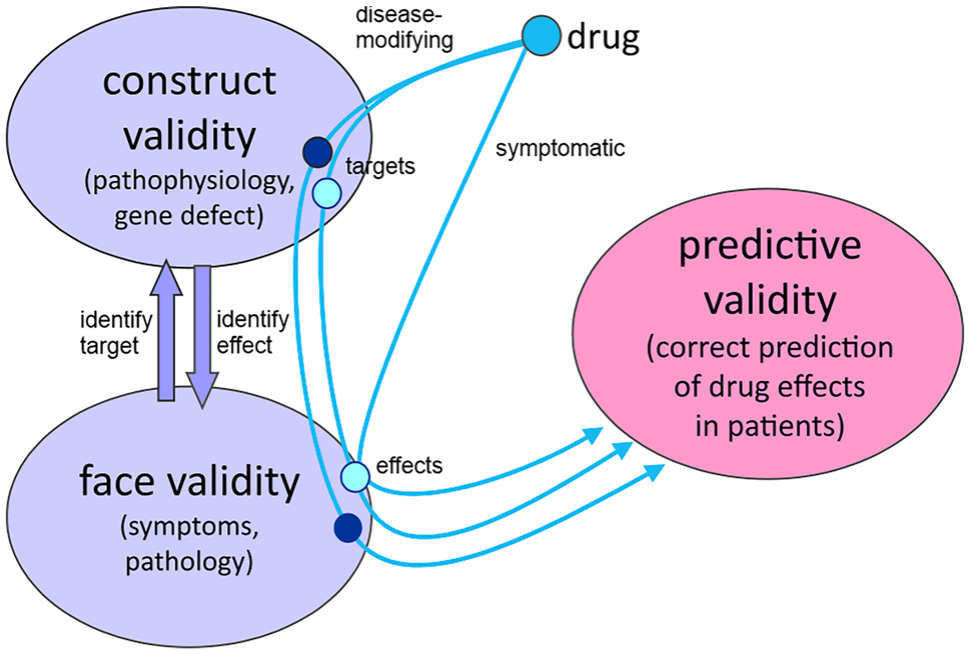
Disease model validity and drug effects. Construct validity describes how closely the make-up of the model replicates etiology and pathophysiology of the disease (e.g., SNCA triplication — overexpression). Face validity describes how the model replicates the down-stream pathology (dopamine loss) and the symptoms (motor, non-motor). Predictive validity describes whether the model can correctly predict drug effects in patients (e.g., established for toxin models for L-dopa symptomatic effects). Neuroprotective/disease-modifying drugs target the disease progression early at the construct, e.g., inhibition of aggregation, while symptomatic drugs target at face validity (dopamine replacement). A single drug may target different pathways/brain regions at early pathophysiology, thereby impacting different symptoms and pathologies (e.g., limbic system versus nigrostriatal system), and may even have an additional symptomatic effect (on- or off-target). Disease-modifying drugs that in addition have symptomatic effects on motor deficits have a higher probability to cross the threshold of sufficient clinical efficacy. It would be useful to detect such multiple effects on readout at the pre-clinical stage. In clinical trials, changes in the brain cannot be directly measured or observed, therefore specific clinical trial designs are necessary to distinguish slowing of disease progression from symptomatic effects. Well-characterized models with defined connections between construct and face validity can help to identify the drug target underlying effects on symptoms and pathology

**Fig. 3:**
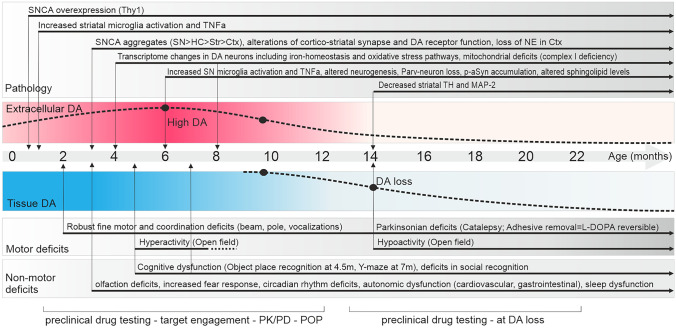
Thy1-aSyn line 61 summary of pathological and behavioral phenotypes developed with age. DA, dopamine (red is extracellular dopamine measured via microdialysis, blue is overall tissue dopamine); PK/PD, pharmacokinetics/pharmacodynamics with exposure–response relationship; POP, proof of principle how a drug with a specific mode of action alters pharmacodynamic responses. Early and progressive pathologies linked to robust functional behavioral readouts allow testing at young ages. Testing closer to age at dopamine loss (14 months) allows measuring delay of striatal neurodegeneration, or pharmacodynamic response under the condition of dopamine depletion. Ctx, cortex; HC, hippocampus; MAP-2, microtubule-associated protein 2; NE, norepinephrine; p-aSyn, phosphorylated alpha-synuclein; SN, substantia nigra; Str, striatum; TH, tyrosine hydroxylase; TNFa, tumor necrosis factor alpha

Models used as final benchmark before moving into clinical trials require highest construct, face, and predictive validity (Fig. 2). While one model is not expected to encompass such a multi-system, multi-faceted, and multi-mechanistic disease, it should at least provide several of each of these aspects, while still reflecting disease mechanisms likely present in most PD patients [19–21]. This includes nigrostriatal dysfunction and motor symptoms to predict main clinical endpoints, but also non-motor symptoms, widespread alpha-synuclein pathology, and inflammatory markers. Here, we provide an update on the Thy1-aSyn (line 61) model reviewed first in 2012 [22] and the considerable progress which has been made over the past decade to optimize its utility for pre-clinical drug testing. First, we will summarize the phenotype previously described with focus on aspects specifically relevant to pre-clinical drug testing (Fig. 3). Second, we will present how the model has been used to uncover disease mechanisms and druggable targets. Third, this is followed by a presentation and discussion of performed pre-clinical trials in Thy1-aSyn mice.

## Thy1-aSyn (Line 61) Model Replicates Alpha-Synuclein Pathology and PD-Like Symptoms

In this chapter, we will summarize previously reviewed characteristics of the Thy1-aSyn line 61 model [22] and complement them with recent discoveries.

### Genetics and Transgene Expression

Mice over-expressing human wild-type *SNCA* (gene encoding for alpha-synuclein) driven by the murine Thy-1 promoter were developed in the laboratory of Eliezer Masliah at UCSD [23] and designated “line 61” [23, 24]. Most laboratories keep this line on a hybrid C57BL/6xDBA2 background by breeding female transgenics with hybrid males purchased from a vendor (transgenic males do not breed well). Females develop less severe behavioral phenotypes compared to makes, which is likely due to the expression of the transgene on the X-chromosome [25]. However, both males and females lose striatal dopamine at 14 months of age [26]. The Thy-1 promoter enables moderate post-natal over-expression of human wild-type alpha-synuclein in all brain regions (including dopamine neurons) and in peripheral neurons, thereby mimicking the expression encountered in sporadic PD and in Lewy body disease in general [23]. This approach is clearly validated (construct validity, Fig. 2) in patients, by (i) the presence of alpha-synuclein accumulation in Lewy bodies [27], a pathological hallmark of synucleinopathies, by (ii) the fact that multiplication of SNCA causes familial forms of PD, and by (iii) the observation that polymorphisms in non-coding regions of SNCA increase PD risk or progression [28–30]. Of note, knock-down of alpha-synuclein in vivo is feasible and ameliorates neurodegeneration [31–34]. Thy1-aSyn mice remain at good general health until overt dopamine loss at 14 months of age, whereby males show a slightly decreased body weight gain starting at 4 months of age, which could be due to motor deficits, circadian rhythm alterations, and/or metabolic changes (see also chapters below).

### Alpha-Synuclein Aggregation

Driven by the Thy-1 promotor, there is widespread accumulation of alpha-synuclein in Thy1-aSyn mice, including the midbrain, cortex, hippocampus, cerebellum, olfactory bulb, and medulla oblongata [22, 35, 36]. Interestingly, progressive accumulation of proteinase K-resistant alpha-synuclein aggregates is particularly noticeable in the entire substantia nigra, with comparably large aggregates present in the pars reticulata (see [37] Fig. 6), which contains a dense network of dendrites from nigrostriatal dopamine neurons. The need to use proteinase K treatment to identify insoluble alpha-synuclein aggregates precludes the identification of their specific location within cells as markers such as tyrosine-hydroxylase (TH) are destroyed by the treatment. Of note, we previously demonstrated accumulation and aggregation of alpha-synuclein in the substantia nigra pars compacta and pars reticulata in this model [22, 37]. In 5-month-old mice, we observed very high accumulation of alpha-synuclein in a subfraction of nigral TH-positive neurons (see [37] Fig. 5). Rockenstein et al. found extensive accumulation of human alpha-synuclein in TH-positive neurons in the substantia nigra of 12-month-old Thy1-aSyn mice (see [23] Fig. 7E). Whether this high accumulation of alpha-synuclein in some neurons, the substantia nigra translates into formation of large aggregates is unclear.

In contrast, large proteinase K-resistant aggregates are not present in the cerebral cortex despite high levels of *SNCA*. Thus, the regionally specific vulnerability to formation of large alpha-synuclein aggregates does not correlate with transgene expression in Thy1-aSyn mice, replicating aspects of specific neuronal vulnerability characteristic for PD. This is very important for pre-clinical studies, as pathology in the substantia nigra and associated motor symptoms represent a main readout for predictive validity of clinical trials. Results from effects on other brain regions and associated behavioral readouts (e.g., hippocampus and cognitive deficits) may not extrapolate to the substantia nigra and motor symptoms.

Formation of aggregated alpha-synuclein in Thy1-aSyn mice appears synchronized with the accumulation of alpha-synuclein phosphorylated at serine 129, which is markedly increased in PD [38]. Again, highest levels are found in the ventral mesencephalon (including the substantia nigra) accumulating in cell somata and nuclei. Neuropil, perikarya, and nuclei staining patterns of human alpha-synuclein differ by brain (sub)region in young (3–4 months) Thy1-aSyn mice. Again, this pathology does not correlate with transgene expression but represents regional vulnerability to alpha-synuclein expression [35]. Some of these pathologies and corresponding behavioral deficits can be replicated and amplified by overexpressing mutated alpha-synuclein prone to oligomerize under the Thy1-promoter [39]. McLean et al. detected accumulation of alpha-synuclein splice variants (SNCA-126, -112, -98) in the PD brains and in 2-month-old Thy1-aSyn mice, again in a regionally specific expression pattern [22, 40]. The most prominent increase of these variants was observed in the ventral midbrain of 15-month-old transgenics (which coincides with overt loss of dopamine in the model), indicating a potential regionally specific pathology [22, 40]. Alpha-synuclein pathology is also observed in the enteric nervous system of PD patients [41, 42] and Thy1-aSyn mice [43], coinciding with the gastrointestinal symptoms observed (see respective chapter below).

### Microgliosis and Inflammatory Markers

In addition, the Thy1-aSyn mice also develop further (downstream) pathologies relevant to the known pathophysiology of PD. Of high relevance to drug testing are the early innate inflammatory reactions in the brain together with peripheral adaptive immune responses [44]. Microgliosis has been connected with various neurologic and neurodegenerative diseases, including PD [45]. Thy1-aSyn mice present with pronounced microglial activation first in the striatum (1 month of age) and later in the substantia nigra (5–6 months of age), together with increased percentages of cytotoxic T cells in the blood at late ages. Tumor necrosis factor alpha (TNFα) and specific Toll-like receptor expression is elevated [46]. Another study using immunohistochemistry revealed an increased number of T cells, especially CD4 + T helper cells, in both Thy1-aSyn and dementia with Lewy bodies brains [47]. Increased intracellular levels of interferon-y suggested a proinflammatory state of T cells. Flow cytometric analyses confirmed these findings, and, additionally, discovered a population of natural killer cells among analyzed cells [47]. A glial-T-cell crosstalk has been proposed previously, providing the basis of glial antigen presentation to T cells, as they need to be activated for acquired immune reactivity (immunological synapse). In the brain of Thy1-aSyn mice, T cells have been found in close proximity to astroglial and microglial cells, supporting the hypothesis of T cell involvement in neurodegeneration. This study also confirmed microgliosis and increase in TNFα expression. There were no differences in IL-2, IL-4, IL-17f, TGFβ, IL-12, and IL-10 levels [47]. Thy1-aSyn mice were crossed with Rag2^−/−^ mice, which lack mature lymphocytes, to assess the role of infiltrating T lymphocytes [48]. Depletion of lymphocytes led to a decreased deposition of alpha-synuclein in substantia nigra and striatum. It appeared that T lymphocytes are aggravating the neuroinflammatory phenotype, mainly by changing myeloid cells phenotype. In the presence of T cells, the phagocytosis of alpha-synuclein by microglia is reduced with concomitant worsening of alpha-synuclein pathology [48].

### Mitochondrial Dysfunction and Oxidative Stress

Deficits of complex-I of the mitochondrial respiratory chain is a signature pathology in PD [49]. Impairment of mitochondrial function is characterized by generation of reactive oxygen species, cytochrome-c release, ATP depletion, and caspase-3 activation [50, 51]. Recent studies uncovered a mechanism of alpha-synuclein-mediated mitochondrial dysfunction. Certain post-translational modifications of alpha-synuclein block the import of proteins into the mitochondria by interacting with the translocase of the outer membrane 20 (TOM20), with detrimental effects on mitochondrial function [52]. Moreover, overexpression of TOM20 was neuroprotective in an AAV2-alpha-synuclein overexpression model of PD [53]. In Thy1-aSyn mice, isolated mitochondria-enriched fractions from the ventral midbrain, striatum, and cortex were examined for alpha-synuclein protein expression [54]. Monomers of alpha-synuclein were more abundant in mitochondrial fraction in all brain regions of Thy1-aSyn compared to control mice. Interestingly, a truncated form of alpha-synuclein (~ 12 kDa) was observed exclusively in mitochondrial fractions of Thy1-aSyn mice. Oligomeric alpha-synuclein was previously found to potently inhibit mitochondrial import [52]. Transgenic mice exhibit an impairment in complex-V in the ventral midbrain and in the striatum together with reduction of basal respiration. Interestingly, decrease in mitochondrial complex-I activity was observed in the ventral midbrain (containing the substantia nigra) only. In this region, there was also an elevation of oxidative stress markers and reactive lipid peroxidation products [54]. Dopamine-modified alpha-synuclein also potently inhibited mitochondrial import via TOM20 [52]. In summary, mitochondrial deficits and downstream toxic events are most prominent in the ventral midbrain of Thy1-aSyn mice, which aligns with alpha-synuclein pathology and microgliosis. Whether this is mediated by reduction in mitochondrial import in Thy1-aSyn mice needs to be determined, but could provide an important druggable target in this model.

### Dopamine Loss and Parkinsonian Phenotypes

Thy1-aSyn mice present with 40% loss of striatal dopamine at 14 months of age [26]. The loss of dopamine in the striatum, a brain region involved in the control of movement, leads to akinesia, rigidity, and potentially tremor in PD [55], represented as hypolocomotion, catalepsy, and reduced sensorimotor response in Thy1-aSyn mice (Fig. 3). In patients and the Thy1-aSyn mouse model, these symptoms are responsive to dopamine replacement therapy [26, 56]. Until this age of overt dopamine depletion, Thy1-aSyn mice represent a model of preclinical PD, particularly useful to study neurodegenerative processes and early therapeutic interventions. At 6–12 months, transgenics present with subtle loss of dopamine transporter, tyrosine hydroxylase, synaptophysin, and PSD95 (postsynaptic marker) expression in the striatum. Interestingly, tonic striatal extracellular dopamine and dopamine metabolites were elevated in Thy1-aSyn mice at this age, accompanied by hyperactivity in the open field [26]. It is unclear whether this reflects processes in sporadic PD. Intriguingly, imaging studies in leucine-rich repeat kinase 2 (LRRK2) carriers also revealed alterations compatible with excess extracellular dopamine, similar to our observations in Thy1-aSyn mice [57]. It has been suggested that alterations in dopamine metabolism represent a burden for dopamine neurons, as dopamine is toxic when it escapes normal regulatory mechanisms [58, 59], especially in conjunction with alpha-synuclein pathology [60]. As extensively discussed previously [22], if examined up to 22 months of age, the loss of dopamine and tyrosine hydroxylase-positive striatal terminals is not followed by loss of dopamine neuron cell bodies in the substantia nigra, even though their diameter was slightly reduced. This resistance to the loss of dopamine cell bodies is common in genetic mouse models of PD [19–21]. The loss of striatal terminals is suggested to precede dopaminergic cell loss in PD [61]; therefore, striatal degeneration in Thy1-aSyn mice together with the behavioral readouts provides a platform to test drugs which modify the underlying pathogenic processes. If neuroprotection is defined as protecting the neuron from degenerative processes, then neurotransmitter and terminal loss represent an important therapeutic target. For substantiating neuroprotective effects with regard to cell body loss, further animal models of PD, such as viral-mediated overexpression of SNCA in the substantia nigra, are available [20]. In Thy1-aSyn mice, a second hit may be required for dopamine neuron loss, as suggested by the “multiple hit” hypotheses of PD [62, 63]. The pesticide paraquat increased alpha-synuclein aggregation in Thy1-aSyn mice, but nigrostriatal dopamine cell loss was about 25%, which is similar to wild-type [64]. Conversely, Thy1-aSyn mice were more sensitive to the combined toxicity of Paraquat and the herbicide Maneb in a study which focused on adult hippocampal neurogenesis [65]. Further studies are needed to understand the gene-environment interaction involving alpha-synuclein accumulation, with attention to susceptibility of different neuronal subtypes.

### Non-Motor Symptoms Reflecting Early PD

There is considerable evidence that in many patients, non-motor symptoms appear before the classical motor symptoms. This may indicate a spread of disease mechanisms from the periphery (gastrointestinal dysfunction) to the brain stem, midbrain, and limbic areas (sleep alterations, neuropsychiatric signs, mild cognitive dysfunction, motor symptoms) and finally to higher brain regions (late cognitive decline), which seems supported by the successive appearance of Lewy pathology along these regions [5, 66–71]. PD pathology may thereby originate in the gastrointestinal tract and only then progress to the brain in some patients, a hypothesis that needs further validation [71–74]. Furthermore, olfactory deficits are frequently observed early in PD [75]. Interestingly, in Thy1-aSyn mice, where *SNCA* expression appears in the PNS and the CNS simultanously, there is still late onset of dopamine loss at 14 months, but early onset of non-motor symptoms (e.g., deficits in olfaction, gastrointestinal dysfunction, sleep alterations, cognitive dysfunction, anxiety phenotype, Fig. 3) [43, 76–82]. These non-motor symptoms can be discriminated in peripherally or centrally induced phenotypes, albeit there is considerable overlap (e.g., involvement of vagal nuclei in gastrointestinal dysfunction).

#### Predominantly CNS Originated Non-Motor Symptoms in Thy1-aSyn Mice

##### Early Cognitive Deficits

Early cognitive deficits are often included as endpoints in drug studies, because they represent a common debilitating symptom unresponsive to dopamine replacement therapy. At 4–6 months of age, Thy1-aSyn mice make fewer spontaneous alternations in the Y-maze and show deficits in tests of novel object recognition (NOR), object-place recognition, and operant reversal learning, compared to wild-type, indicative of impairments in exploratory behavior, spatial learning, and recognition memory [78]. Magen et al. [79] also identified social cognitive impairments in Thy1-aSyn mice. Thy1-aSyn mice at ages of 7–8 months spent less time exploring social partners without differences in exploration of inanimate objects. This study highlights that complex phenotypes require careful testing and controls to restrict measurements to a specific deficit, because the olfactory, cognitive, and motor deficits will influence outcome of any behavioral test. The investigator needs to be aware of all currently known phenotypes and their time points of expression (Fig. 3), in order to design therapeutic drug studies and interpret behavioral endpoints.

##### Impairments in Sleep and Network Excitability

Of increasing importance among centrally originated non-motor symptoms are impairments in sleep, especially given the increased risk of patients with REM sleep behavioral disorder to develop PD [83]. McDowell et al. [80] employed non-tethered EEG/EMG (electroencephalogram/electromyography) telemetry recordings during different vigilance states including wakefulness, non-REM sleep, and REM sleep in Thy1-aSyn mice. During the active phase, Thy1-aSyn mice spent more time in active wake, entered REM phase less often, and spent less time in REM sleep phase. Sleep/wake architecture of the Thy1-aSyn mice was analyzed further and no significant differences in the number of bouts or mean length for active wake or quiet wake phases were observed. Interestingly, in Thy1-aSyn mice, sleep onset latency (during the active phase) and non-REM sleep bout length were increased when compared with wild-type mice, while sleep fragmentation analysis showed decrease in the number of awakenings from sleep in Thy1-aSyn mice when compared to wild-type. Relative power density analysis between each vigilant state showed a shift in the density of their power spectra toward lower frequencies depicted in increase in delta and theta power and a decrease in gamma power during bouts of either active wake or quiet wake. During non-REM sleep, Thy1-aSyn mice showed an increase in delta power and a decrease in alpha power when compared to wild-type mice [80].

We have recently replicated this phenotype (which was first described by the Chesselet lab at UCLA) and observed alpha-synuclein pathology and microgliosis in the brain regions relevant to sleep regulation (unpublished results). We could also confirm findings of epileptiform activity in parallel to slowing of brain oscillations, previously discussed as relevant to synucleinopathies [84]. In the barrel cortex, Thy1-aSyn mice exhibit augmented, long-lasting calcium transients characterized by considerable deviation from the exponential decay observed by 2-photon microscopy [85]. Altered response to repetitive stimulation indicated interference of alpha-synuclein with intracellular calcium buffering mechanisms. Neuronal cell type classification showed no differences in the ratio of inhibitory to excitatory neurons and there was no increase in neuronal spiking response compared to wild-type. A follow-up study used the calcineurin inhibitor FK506 to normalize calcium transients in Thy1-aSyn mice [86], as a proof that this endpoint and 2-photon microscopy may be a suitable bioassay for compound testing. Further studies are warranted to better discriminate sleep alterations from abberrant network excitability, or to understand a potential relationship, respectively. Furthermore, studies are required to determine whether sleep dysregulation is aggravating the phenotype progression in Thy1-aSyn mice, which would support early therapeutic intervention in PD.

##### Abnormalities in Energy Metabolism

Non-motor symptoms of PD include also metabolism abnormalities, weight loss, and energy expenditure changes that are centrally regulated [87–89]. As mentioned above, Thy1-aSyn mice display lower weight gain compared to wild-types as early as at 3 months of age, which could be related to the below described gut dysfunction. Interestingly, a recent study determined that Thy1-aSyn mice develop no difference in lean mass but reduced body fat [90]. The authors examined food intake, feeding patterns, ambulatory activity, and energy expenditure using indirect calorimetry system. Thy1-aSyn consumed less food during dark (active) phase and were significantly more active in both dark and light phases of the day, confirming previously described hyperactivity in young mice. Finally, the authors observed that temporal rhythms in energy metabolism might be dysregulated since Thy1-aSyn mice did not show reduction in oxygen consumption in light (inactive) phase as wild-types do. Furthermore, Thy1-aSyn mice have decreased insulin and leptin plasma levels, while no significant changes in glucose or insulin responses were observed. Accumulation of alpha-synuclein, phosphorylated alpha-synuclein (Ser129), and aggregated alpha-synuclein were found in the hypothalamus, the brain region with a central role in the regulation of metabolic processes (e.g., feeding, energy expenditure). Pathways known to couple hormonal signals with the maintenance of metabolic and energy homeostasis were impaired in the hypothalamus, potentially underlying the disruption of feeding regulation and energy metabolism in Thy1-aSyn mice [90].

#### Predominantly PNS Originated Non-Motor Symptoms in Thy1-aSyn Mice

Wang et al. observed alterations in colonic myenteric ganglia and defecation in Thy1-aSyn mice [43, 81]. Reduction of fecal output in response to novelty stress and post-prandially was observed in young (2.5–3 months) and older (8 months) Thy1-aSyn mice. Immunohistochemistry of distal colonic myenteric neurons in Thy1-aSyn mice revealed increased expression of alpha-synuclein, mainly localized to varicosities of neuronal fibers in distal colon myenteric plexuses with a higher density of alpha-synuclein positive fibers in distal colonic myenteric ganglia compared to wild-type. Proteinase K-resistant aggregates could not be detected. The authors observed no difference in morphology of both nNOS- and pChAT-positive myenteric neurons of the distal colon between wild-type and Thy1-aSyn mice; however, alpha-synuclein-positive varicose terminals were adjacent to the pChAT-positive neurons. Ongoing studies have verified that alpha-synuclein derived from the gastrointestinal system of Thy1-aSyn mice can seed formation of aggregation in brain tissue lysate and vice versa (Zunke and Richter, unpublished results). Hence, in addition to the impact of local SNCA expression in peripheral and central neurons, spread of pathology could be present in Thy1-aSyn mice, which is supported by experimental studies altering the gut microbiome [91] (see respective chapter below).

Hallett et al. investigated presence of internal organ pathology and autonomic deficits in Thy1-aSyn mice [92]. Gross abnormalities were observable already in 2-month-old Thy1-aSyn animals. Intestinal tract was of different pallor and size, and showed distinct areas of enlargement. The urinary bladder was enlarged in 9-month-old Thy1-aSyn mice. Fecal boli production was reduced at 9–12 month in Thy1-aSyn mice, the fecal pellets were drier, and the whole gut transit time was increased [92]. While food and water intake did not differ in this study, the previously described lower weight gain was replicated. Histological assessment of the gastrointestinal system of 15-month-old mice revealed structures resembling axonal swellings that were immunoreactive for human alpha-synuclein in Thy1-aSyn mice. Small insoluble alpha-synuclein aggregates were observed as well in this study [92]. Alpha-synuclein immunoreactive fibers and occasional cell bodies were detected in the myenteric plexus. Alpha-synuclein immunoreactive fibers were also present in the submucosal plexus and in intestinal villi. Histological assessment showed rare co-localization of alpha-synuclein with TH-, vesicular acetylcholine transporter (VAChT)-, and vasoactive intestinal peptide (VIP)-positive neurons. Human alpha-synuclein puncta were located near but not in neuronale NO-synthase (nNOS)-immunoreactive neurons. Human alpha-synuclein was not detected in the dorsal motor nucleus of vagus of Thy1-aSyn mice, but there was abundance of human alpha-synuclein in the hypoglossal nucleus [92]. Histological assessment of the cardiac system revealed proteinase K resistant aggregates in the ventricle and atrial walls of the Thy1-aSyn mice, and overexpression of human alpha-synuclein was detected in noradrenergic neuronal fibers. Finally, human alpha-synuclein expression was also detectable in the skin of the Thy1-aSyn mice [92].

In an effort to characerize further non-motor symptoms related to potential peripheral denervation, Fleming et al. investigated baroreflex function in Thy1-aSyn mice [93]. Baroreflex failure is documented in patients with PD [94] and is often considered the root cause for the observed orthostatic hypotension. While systolic blood pressure and heart rate did not differ, Thy1-aSyn mice showed a blunted heartrate response to sodium nitroprusside and atropine indicating deficits in baroreflex function [93]. This phenotype should also be kept in mind for invasive studies requiring surgery, as we find transgenic mice to be more sensitive to anesthetics and surgical interventions (decompensation of cardiovascular system).

### Recruiting Early Neuronal Dysfunction as Drug Study Endpoints

#### Early Motor Deficits as Sensitive Readout for Neuronal Dysfunction

Symptoms appear if neuronal circuits cannot compensate sufficiently for dysfunction, which depends on intrinsic propensities of neuronal subtypes and networks. With regard to pre-clinical drug testing in Thy1-aSyn mice, specific symptoms should be carefully characterized and linked to underlying dysfunction of specific neuronal subtypes, to be useful surrogates for successful target engagement. In PD patients, Lewy pathology in specific neurons appears to correlate with motor and non-motor dysfunction, e.g., in the gastrointestinal tract with constipation, in midbrain neurons with motor symptoms, and in cortico-limbic regions with neuropsychiatric signs and cognitive decline [5, 66–71]. Our insights from Thy1-aSyn mice demonstrate that alpha-synuclein accumulation is sufficient to induce these specific functional deficits. Here, subregional alpha-synuclein pathology appears at ages when corresponding phenotypes are developed such as in the olfactory bulb (olfaction deficits) [76], the basolateral nucleus of the amygdala (fear and anxiety circiutry) [82], and the nigrostriatal system (motor symptoms) [26]. Thereby, the sensitivity of the employed tests is crucial: Sheila Fleming and others could observe progressive deficits in motor tests at 1–2 months of age in Thy1-aSyn mice, such as a beam overlaid with a grid, the pole test, the nest building test, or when carefully observing limb movements in a cylinder [36, 95]. Using these tests, the motor phenotype is robustly and strongly expressed, providing a wide dynamic range for improving or worsening drug effects. Similarly, carriers of the LRRK2 mutation, a frequent cause of familial PD, display early deficits in challenging motor tests, before the onset of parkinsonian symptoms [96]. The number of “errors per step” on the challenging beam is currently the most reliable and robust measure to detect drug effects on motor function with high power [22] and should be included in all pre-clinical trials in Thy1-aSyn mice.

More recently, we characterized ultrasonic vocalization deficits as another very specific and early motor phenotype in the Thy1-aSyn mice, thereby replicating speech deficits in patients [97]. Thy1-aSyn mice vocalized less frequently as they aged with an altered call profile and reduced intensity range at 6–7 months of age, coinciding with alpha-synuclein pathology in the periaqueductal gray nucleus, which is implicated in the coordination of vocal behaviors [97].

#### Reverse-Engineering for Mechanistic Insights from Pre-Clinical Trials

Notably, in pre-clinical studies, each drug effect directly linked to a mechanistic target (e.g., enhanced lysosomal function [37]) increases the knowledge on which pathophysiology causes a specific phenotype, and whether the symptoms and/or their progression are amenable to modifications of a specific pathway. Therefore, pre-clinical trials serve to (reverse-)engineer the validity of the model, tightening the mechanistic link between construct and face validity (Fig. 2). Of note, this concept requires exquisite understanding of the drugs mode(s) of action and does not represent an early screening or high-throughput approach. A simplistic example is the observation that administration of L-dopa or dopamine agonists at an age of high extracellular striatal dopamine worsens motor deficits in Thy1-aSyn mice [98], in contrast to the later deficits that are improved by the dopamine replacement [26]. As discussed previously, excess dopamine on top of the dysfunctional but not yet deficient nigrostriatal dopaminergic system in Thy1-aSyn mice and in patients is worsening symptoms [22, 99]. Therefore, if a compound is increasing these early motor deficits, follow-up analysis has to determine whether this was caused by a symptomatic increase of dopamine signaling, or whether it reflects a (selective) disease-worsening effect on the pathology present in the nigrostriatal system of Thy1-aSyn mice. We have experienced both scenarios in drug trials ran in this model, worsening of the errors per step on the challenging beam test coinciding with increased expression of dopamine-related transcripts [99], and worsening of symptoms coinciding with increased pathology specifically in the substantia nigra in a pre-clinical study testing venglustat (glucosylceramide synthase inhibitor to improve lysosomal alpha-synuclein degradation) (presented at AD/PD 2022; manuscript in revision). Venglustat was tested in parallel in phase 2 (MOVES-PD) and worsened motor symptoms in PD patients. Hence, knowledge on how behavioral readouts in Thy1-aSyn mice respond to pharmacological interventions allows conclusions that are more informative in pre-clinical trials.

## Thy1-aSyn Mice as Useful Tool to Discover and Validate Novel Drug Targets

In the following chapters, we will highlight some findings from selected in vivo studies between 2012 und 2020 using Thy1-aSyn mice to study pathophysiology of PD, including proof of concept pharmacological interventions. This does not serve to present all available studies and data, but to demonstrate the utility of the model for discovery and validation of novel drug targets.

### Protein–Protein Interactions and Post-Translational Modifiers of Alpha-Synuclein

Interestingly, in dementia with Lewy bodies patients and in Thy1-aSyn mice, co-localization of p38γ mitogen-activated protein kinase (MAPK) was intimately associated only with intracellular alpha-synuclein aggregates located in neuronal cell bodies. The authors confirmed interaction between alpha-synuclein and p38γ in vitro [100]. A role of p38 MAPKs in PD is intriguing, since they are involved in regulation of processes such as cell death, oxidative stress, inflammation, and phosphorylation of alpha-synuclein [101, 102]. Apoptosis signal-regulating kinase 1 (ASK1) is activated by oxidative stress and inflammation and is an upstream regulator of c-Jun N-terminal kinases (JNK) and p38 MAPK. In Thy1-aSyn mice, levels of phosphorylated ASK1 were increased in the cortex [103]. Double transgenic animals (Thy1-aSyn/ASK1-/-) present with lower levels of microgliosis in the cerebral cortex restored levels of calbindin in the hippocampus and improvement of motor deficits. However, there was no effect on alpha-synuclein aggregation or phosphorylation in Thy1-aSyn mice [103]. Mutations in the gene superoxide dismutase 1 (SOD1) are causative for familial forms of the neurodegenerative disease amyotrophic lateral sclerosis. Interestingly, SOD interacts with alpha-synuclein with some evidence of promotion of oligomerization, an association also observed in Thy1-aSyn mice [104].

Alpha-synuclein oligomers proved to be toxic in PD and other synucleinopathies [105]. Ferreira et al. found that oligomeric species of alpha-synuclein form a complex with cellular prion protein (PrP^C^) which mediated by metabotropic glutamate receptor 5 (mGluR5), activated NMDA receptors, and altered calcium homeostasis [106]. Blockage of these pathways in Thy1-aSyn mice rescued synaptic and cognitive deficits, supporting their utility for testing drugs that target oligomer-mediated alpha-synuclein pathology [106]. Overk et al. generated double transgenic mice over-expressing amyloid precursor protein (APP) and alpha-synuclein (APP/Thy1-aSyn mice) and investigated the relationship between alpha-synuclein cleavage, Aβ, mGluR5, and neurodegeneration in the hippocampus [107]. Knock-down of mGluR5 via lentivirus sh-RNA protected hippocampal neuronal cells from the neurotoxic effects of Aβ and alpha-synuclein. In vitro, Aβ promoted calpain-mediated alpha-synuclein fragmentation and caspase-3 dependent cell death, which was replicated in vivo in APP/Thy1-aSyn mice [107]. Other studies using conformation specific antibodies confirmed the presence of C-terminally cleaved alpha-synuclein in the brains of Thy1-aSyn mice with prominent staining of neuropil and dystrophic neurites [108].

Transglutaminase 2 (TG2) is implicated in PD, as well as in other neurodegenerative diseases characterized by protein misfolding [109]. TG2 promotes formation of high molecular weight alpha-synuclein aggregates in vitro and in vivo, including in Thy1-aSyn mice [110, 111]. TG2^KO^/Thy1-aSyn double transgenics presented with less alpha-synuclein pathology and microgliosis, increased microtubule-associated protein 2 (MAP2) immunoreactivity demonstrating nerve terminal rescue, and reduction in motor impairments [112]. Glycation and formation of advanced glycation end-products (AGE) are suggested to play a role in PD [113]. Methylglyoxal (MGO), a toxic metabolite of these pathways, was injected intracranially in Thy1-aSyn mice [114]. MGO exposure increased alpha-synuclein glycation and accumulation, reduced the number of dopaminergic neurons, and caused neuritic degeneration in the striatum. Alpha-synuclein glycation was confirmed in the human brains. MGO promotes alpha-synuclein oligomerization, impairs its lipid binding, and interferes with its clearance, thereby presenting a drug target [114]. Thy1-aSyn mice were also used for in vivo demonstration that c-Abl tyrosine kinase, an indicator of oxidative stress, phosphorylates alpha-synuclein, and regulates its degradation [115]. Alpha-synuclein phosphorylated at Y39, the primary residue phosphorylated by c-Abl in vitro, accumulates in the brains of PD patients and Thy1-aSyn mice. Therefore, Thy1-aSyn mice are suitable to study therapeutic strategies targeting c-Abl [115].

### Expression Regulators of Alpha-Synuclein and Downstream Expression Changes

Overexpression of wild-type alpha-synuclein by gene multiplication is causing disease in a dosage dependent manner (triplication leads to more severe and earlier onset disease than duplication) [116]. Nigral dopaminergic neurons of sporadic PD patients express about 7-fold higher levels of alpha-synuclein mRNA [117]. Specific reduction of gene expression can be achieved by using synthetic non-coding small interfering RNAs (siRNA). Packaging of siRNA into nanoparticles increases stability, distribution, and transfection rate which allows injection into cerebral spinal fluid with widespread protein knock-down in the brain evident 5 days after a single application [34]. Rousseaux et al., using fruit fly and mouse models as well as human *post mortem* samples, identified that tripartite motif-containing 28 protein (TRIM28) upregulates alpha-synuclein (and Tau) expression [118]. Thy1-aSyn mice were used to test if reduction of TRIM28 expression can ameliorate age-dependent accumulation and aggregation of alpha-synuclein by crossing the line with TRIM28 + / − mice. Reduced TRIM28 expression resulted in significant reduction of pathological phosphorylated alpha-synuclein accumulation while increasing TRIM28 expression using a lentiviral system worsened neuropathology in Thy1-aSyn mice. TRIM28 is elevated in the PD brains, thus presenting a promising drug target, which is also responsive in Thy1-aSyn mice [118]. Downregulation of doublecortin-like kinase 1 (DCLK1), a microtubule binding serine threonine kinase, reduces alpha-synuclein expression post-transcriptionally [119]. Knock-down of DCLK1 by neonatal intraventricular injection of Adeno-associated virus 8 (AAV8)-shDCLK1 in Thy1-aSyn mice resulted in reduction of phosphorylated alpha-synuclein present in the soma [120].

We previously highlighted extensive gene expression changes caused by alpha-synuclein over-expression [22, 99]. Physiologically, alpha-synuclein is localized in the nucleus, and the accumulation of phosphorylated and aggregated alpha-synuclein in nuclei of Thy1-aSyn mice was observed by us and by others [121]. These authors also observed alpha-synuclein, pS129 alpha-synuclein as well as aggregated alpha-synuclein in the nucleus in the brains of synucleinopathy patients [121]. Thus, nuclear accumulation of alpha-synuclein pathology in Thy1-aSyn mice warrants more attention. The proteome of brain samples from Thy1-aSyn mice was characterized to reveal PD related pathways in 17 distinct neuroanatomical regions of the adult mouse brain via state of the art mass spectroscopy technologies [122]. Overexpression of human alpha-synuclein-induced region and genotype specific increase in extracellular matrix remodeling and inflammation markers (e.g., Hspg2, Lama5, and Lamb2) by 7 months in Thy1-aSyn substantia nigra, and several proteins were suggested as potential therapeutic target [122].

### Role of Lipids, Lysosomal Function, and Autophagy in Alpha-Synuclein Pathology

Depletion of intraneuronal lysosomes, accumulation of undegraded autophagosomes, and decreased levels of lysosomal-associated proteins are detected both in human patients and in animal models [123, 124]. Loss of function of ATPase 13A2 (ATP13A2, PARK9) disrupts lysosomal polyamine export and is associated with neurological conditions including PD [125]. Thy1-aSyn crossed with Atp13a2 knockout mice displayed earlier and more progressive behavioral abnormalities compared to their parental genotypes [126], confirming the expected detrimental association of lysosomal dysfunction and alpha-synuclein accumulation. Lysosomal dysfunction in Thy1-aSyn is also apparent in age-dependent alpha-synuclein immunoreactive axonal swellings (“globules”) in various brain areas [127]. Interestingly, globules were immunopositive for several GABAergic markers suggesting that they originated from GABAergic neurons. Ultrastructural analysis of alpha-synuclein globules uncovered membranous elements, autophagosome-like structures, multivesicular bodies, and multilayered membranes. Furthermore, globules were immunopositive for gangliosides, which together suggested involvement of autophagy-lysosomal system in their formation in Thy1-aSyn mice. Immunoelectron microscopy showed accumulation of mitochondria with potentially damaged outer membrane in alpha-synuclein globules and increased oxidative stress markers in the brains of Thy1-aSyn mice [127]. Interestingly, LRRK2 protein (causing familial PD) associated with alpha-synuclein globules, while Parkin, PINK1, and DJ1 were not observed [127].

Ubiquitin carboxyl-terminal hydrolase L1 (UCH-L1) inhibition was proposed to alter alpha-synuclein pathology, whereby in PD mutations in this protein may be detrimental (familial forms) or reduce risk [128, 129]. UCH-L1 inhibition in Thy1-aSyn mice reduced mono-ubiquitin levels and (only in transgenics) exerted significant reduction of alpha-synuclein accumulation in the hippocampus likely via promotion of autophagy [130]. Mutations in the gene for glucocerebrosidase (GBA1) are causative for the lysosomal storage disorder Gaucher Disease, but also represent a major risk factor to develop PD [131–133]. Therefore, increasing glucocerebrosidase activity is a rational therapeutic target [134]. Rocha et al. tested the potential of GBA1 overexpression to reduce alpha-synuclein pathology by intracerebral injection of AAV-GBA1 in Thy1-aSyn mice [135]. In targeted structures (striatum, substantia nigra, hippocampus), GBA1 overexpression caused the expected increase in glucocerebrosidase activity. Interestingly, reduction of alpha-synuclein pathology was evident only in striatum and substantia nigra, again pointing towards regionally specific pathomechanisms [135]. Cell transplantation studies in Thy1-aSyn mice revealed that knock-out of glucocerebrosidase in the transplant increases the deposition of host alpha-synuclein into the transplanted cells. Hence, lysosomal dysfunction is rendering neurons more vulnerable to the prion-like spread of alpha-synuclein pathology [136].

Thy1-aSyn mice were also used to study the role of the ESCRT-III (endosomal sorting complex required for transport III; proteins involved in the sorting and trafficking of proteins from the endosome to the lysosome via the formation of multivesicular bodies) complex in alpha-synuclein-associated pathology [137]. Transgenic mice mimicked alterations in ESCRT-III composition and their interaction with alpha-synuclein found in synucleinopathy patients. Thy1-aSyn mice that received lentivirus-mediated increase of specific ESCRT-III components in the hippocampus showed a significant reduction in the accumulation of alpha-synuclein in neuronal cell bodies and neurites, reduced neurodegeneration (NeuN) and GFAP immunoreactivity, and reduction of hyperactivity and memory deficits (context dependent learning in the open field) [137].

### Mechanisms of Oxidative Stress and Mitophagy Involving Alpha-Synuclein

The 10 kDa heat shock protein (HSP10) was identified as a mediator of alpha-synuclein-induced mitochondrial impairments in striatal synaptosomes of Thy1-aSyn mice [138]. HSP10 is a component of the major folding complex in mitochondria and it is involved in respiration, membrane potential, and reactive oxygen species removal [138]. The authors showed that alpha-synuclein requesters HSP10 from the mitochondria into the cytosol, thereby leading to a toxic loss of function. Importantly, these alterations appear to manifest first at presynaptic terminals. Overexpression of HSP10 in Thy1-aSyn mice ameliorates alpha-synuclein-associated mitochondrial dysfunction in synaptosomes and delays pathology [138].

Nitrosative stress may also be causative for mitochondrial dysfunction. In Thy1-aSyn mice, S-nitrosylated PINK1 was detectable at early age, which leads to inhibition of PINK1 kinase activity and prevention of mitophagy, and ultimately cell death [139]. Transient receptor potential canonical 3 (TRPC3) channel is the only member of the group of non-selective calcium channels localized in mitochondria, where it plays a role in maintaining the normal mitochondrial membrane potential [140]. In Thy1-aSyn mice, age associated increase in TRPC3 expression was observed in mitochondrial fractions of striatum and cerebellum (no other regions determined). In vitro experiments revealed that high TRPC3 reduces mitochondria membrane potential and increases apoptotic cell death [140].

### Influence of the Microbiome on Alpha-Synuclein Pathology

In recent years, bidirectional communication between the brain and gut is implicated in neurological disorders [141–143]. Sampson et al. investigated the role of gut microbiota in motor deficits, microglia activation, and alpha-synuclein pathology using Thy1-aSyn mice [91], thereby confirming the hypothesis that gut bacteria regulate both motor impairment and alpha-synuclein-induced pathology. Interestingly, the authors observed delay in motor phenotype, reduction in gastrointestinal dysfunction, reduction of alpha-synuclein aggregation, and less microglia activation in Thy1-aSyn mice derived in germ-free conditions (without microbiome). This effect was replicated by treating mice under specific pathogen free conditions (with microbiome) with antibiotics. Treatment with short-chain fatty acids, which are microbial metabolites previously proposed as beneficial, promoted regional specific microglial activation in both wild-type and Thy1-aSyn mice, and alpha-synuclein aggregation and motor impairment in Thy1-aSyn mice. Sensorimotor test performance in Thy1-aSyn mice kept without microbiome and transplanted with microbiota derived from PD patients, significantly deteriorated when compared to both Thy1-aSyn mice with microbiota from healthy subjects and wild-type mice. Interestingly, PD microbiota did not induce motor deficits in wild-type mice [91]. Apart from highlighting the importance of microbiome for progression and symptoms of PD, this study should also raise awareness for housing conditions of experimental animals. The development of a microbiome is highly relevant to disease mechanisms and the stable phenotype expression in Thy1-aSyn mice.

A recent study demonstrated decreased levels of duodenal glucocerebrosidase production in Thy1-aSyn mice [144]. As explained above, reduced activity of this enzyme signifies lysosomal deficits, which augments alpha-synuclein pathology and represents a major risk factor for PD. However, such considerations were focused on the brain, not the gut. Neuronal expression of glucocerebrosidase using a viral vector system partially rescued the gastrointestinal phenotype and the enteric nervous system network connectivity deficits observed in Thy1-aSyn mice. Of note, in this study, dopamine loss was already apparent at 12 months of age in Thy1-aSyn mice [144].

## Pre-Clinical Testing of Prospective Neuroprotective/Disease-Modifying Compounds

Here, we will describe selected studies that were testing compounds for beneficial effects in Thy1-aSyn mice published between 2012 and 2022. Disease models are required to test in vivo target engagement, pharmacodynamic responses, and functional efficacy endpoints to establish proof of principle. There is no perfect model that represents all features of PD, but the model selected for a specific drug trial should obviously harbor the pathomechanism which is targeted (e.g., aggregation of alpha-synuclein), and provide a set of related pathological and behavioral readouts to measure exposure response of drug efficacy [19]. The strategies are grouped by target. PD represents the most frequent synucleinopathy [145], and reduction of alpha-synuclein pathology is a key therapeutic target for disease-modifying strategies [10, 146].

### Lowering of Alpha-Synuclein Expression

If alpha-synuclein accumulation and Lewy body formation is believed to correlate with neuronal dysfunction and PD symptoms, lowering alpha-synuclein expression is a rational therapeutic approach. Strategies aim at reducing transcription of the *SNCA* gene through epigenetic modifications or transcription factors and at reducing translation of *SNCA* mRNA via nucleic acid–based therapeutics or small molecules [10, 146]. In proof of concept studies in Thy1-aSyn mice, we demonstrated that intracerebroventricular administrations of siRNA or antisense oligonucleotides against SNCA effectively reduce alpha-synuclein accumulation and down-stream pathology, as well as behavioral deficits [34, 147].

### Alpha-Synuclein Assembly and Aggregation Inhibitors

#### Inhibition of Oligomer Formation

Given the self-assembly of alpha-synuclein into toxic species, stabilizing the protein in its physiologic non-toxic soluble form represents a rational target. Small molecules that cross the blood–brain barrier and interact with alpha-synuclein were shown to improve behavioral, neuropathological and biochemical endpoints in preclinical trials in Thy1-aSyn mice [148, 149]. The molecular tweezer CLR01 was chosen because it shields lysine (Lys) residues with low affinity, thereby interrupting formation of oligomers without interfering with the physiological protein function. It has been tested as novel drug candidate in several models for amyloidosis, including in Thy1-aSyn mice [148]. CLR01 administration by continuous intracerebroventricular infusion for 28 days in young Thy1-aSyn mice did not affect the appearance of protein aggregates in the substantia nigra, but reduced striatal alpha-synuclein protein in the buffer soluble fraction. Interestingly, CLR01 increased homogenous staining of cytoplasmic alpha-synuclein in dopaminergic neurons of the substantia nigra in Thy1-aSyn mice and robustly improved motor behavior [148]. Follow-up studies in other models of PD and in human dopaminergic cultures revealed neuroprotective effects [150].

NPT100-18A was developed de novo by molecular modeling methods targeting a C-terminus domain of alpha-synuclein, which is important for dimerization and membrane penetration [149]. As expected, the compound reduces binding of alpha-synuclein to membranes, which, however, could represent an important physiological mechanism, at least in synaptic vesicles. Regardless, the compound is highly effective in reducing alpha-synuclein pathology in frontal cortex, hippocampus, and striatum albeit not in the substantia nigra, without overt side effects [149]. Interestingly, reduction of membrane-bound alpha-synuclein coincided with increase in cytosolic protein. NPT100-18A administration over several months in young Thy1-aSyn mice reduced motor phenotype on the challenging beam and other behavioral tests [149]. The authors discussed that the compound prevents toxic interaction of alpha-synuclein with the plasma membrane and other intracellular organelles, without altering the physiological form of alpha-synuclein-associated with synaptic vesicles [149]. NPT100-18A had limited oral bioavailability and relatively poor brain penetration; therefore, subsequent candidates were developed by lead optimization efforts. NPT200-11 (aka UCB0599) is orally bioavailable and brain penetrating while retaining the positive effects on pathological and behavioral readouts after 3 months treatment of Thy1-aSyn mice, including markers of neuroprotection (e.g., increase of dopamine transporter expression in the striatum) [151].

#### Inhibition of Alpha-Synuclein Phosphorylation

As observed in PD and in dementia with Lewy bodies patients, methylation of protein phosphatase 2A (PP2A), a process crucial for dephosphorylation of alpha-synuclein, is reduced in Thy1-aSyn transgenics [152]. Phosphorylated alpha-synuclein is associated with the aggregated protein and with Lewy bodies; therefore, dephosphorylation is thought to reduce toxic protein aggregation. Eicosanoyl-5-hydroxytryptamide (EHT) maintains the methylated state of PP2A and thereby dephosphorylates alpha-synuclein. EHT occurs naturally in coffee beans and was previously shown to be neuroprotective in Thy1-aSyn mice [153]. As a follow up, it was tested at lower dosage together with caffeine that itself has shown some neuroprotective properties for synergistic effects in Thy1-aSyn mice [152]. Caffeine treatment does not improve any observed behavioral parameters while low dose EHT treatment improved performance only in the wire hang test. Combined treatment, however, improved performance in wire hang test, rotarod, nesting behavior, and Morris water maze. Furthermore, caffeine/EHT co-treatment reduced phosphorylated alpha-synuclein and protected against neuronal damage and neuroinflammation [152].

#### Challenges in Therapeutic Development Targeting Aggregation

The outcome of targeting potentially toxic post-translational modifications [154] and the complexity of alpha-synuclein oligomerization and aggregation into higher order species is difficult to predict from in silico or in vitro studies. Upon interaction with such compounds, alpha-synuclein species may redistribute to subcellular compartments or associate with membranes, they may be packaged in higher order aggregates, which could be more or less toxic, or may be released by neurons to be taken up by other cells, to name a few of many possible scenarios. Cytosolic alpha-synuclein in specific neurons or in different subcellular fractions prepared from tissue lysate may therefore increase or decrease, and aggregates may change in size and number. Without well-defined functional behavioral and additional pathology readouts, these observations cannot be interpreted as beneficial or detrimental. Direct interference with higher order aggregates could be deleterious, because increase in the concentration of lower molecular weight assemblies may produce toxic strains, even if the compound appears to decrease the size of aggregates. Thus far, in our hands, only knock-down of alpha-synuclein via antisense oligonucleotide or siRNA in young Thy1-aSyn mice has overtly decreased all alpha-synuclein related readouts in all analyzed brain regions, together with improvement of CNS originated behavioral deficits and reduction of microgliosis [147]. However, this demands complex drug delivery and there is ongoing discussion on a detrimental loss of function of alpha-synuclein aggravated by this strategy. Apart from these considerations, it is not yet clear whether targeting the initial process of alpha-synuclein aggregation can improve PD symptoms at a point where there is already extensive Lewy body pathology.

### Antibodies to Inhibit Spread of Alpha-Synuclein Pathology

Transfer of misfolded alpha-synuclein and thus propagation of pathology across the brain is targeted via active (alpha-synuclein mimicking peptides) and passive (antibodies against human alpha-synuclein) immunotherapies. Thy1-aSyn mice accumulate disease-relevant C-terminally cleaved alpha-synuclein in neurons and neuropil [108], and several antibodies against the C-terminus have been tested in Thy1-aSyn mice [155, 156], including prasinezumab (RO7046015) which is far advanced in clinical trials [157]. The murine form of prasinezumab (9E4; syn aa 118–126) was administered weekly intraperitoneally over 5–6 months and showed reduced neuronal and synaptic loss and a reduction in intraneuronal build-up of alpha-synuclein pathology, reduction of gliosis, and an improvement in both cognitive and motor behaviors. Interestingly, these studies used 6-month-old female Thy1-aSyn mice and the water maze as mixed cognitive and motor readout, while most studies use male mice. Probably due to the insertion of *SNCA* on the X-chromosome, females have delayed and more variable progression of some pathologies and of the behavioral phenotype. We found that in male transgenics, the motor deficits interfere with interpretation of cognition in the Morris water maze (mice do not swim), prompting us to use novel object recognition and Y-maze tests that depend less on motor control. However, for testing 9E4, behavioral tests (round beam and Morris water maze) were only done at the end of treatment period, at approximately 12 months of age, and pathology and behavioral deficits were sufficiently developed in females (group size *n* = 14 was sufficient to detect drug effects) [155, 156]. 9E4 clearly reduced alpha-synuclein pathology and gliosis and increased striatal synaptic markers demonstrating neuroprotective properties [155]. Further efforts were done to increase brain-penetration and to target antibodies against specific conformational forms of alpha-synuclein, with Thy1-aSyn mice as first in line model to test efficacy, with considerable success [158, 159]. Given that these antibodies act extracellularly, their efficacy in Thy1-aSyn mice supports considerable release and prion-like spread of toxic species in this model, contributing to progression of pathology and behavioral deficits. In that sense, the model may be closer to the pathophysiology in sporadic PD than injections of pre-fibrillated protein into the brain of alpha-synuclein over-expressing mice via stereotactic surgery.

### Enhancement of Lysosomal Degradation of Alpha-Synuclein

As already described above, lysosomal dysfunction is regarded as a central pathomechanism in PD. A feed-forward pathological loop between mutant lysosomal glucocerebrosidase loss of function and alpha-synuclein accumulation could underlie the increased risk for PD among carriers of mutant *GBA1* alleles [160–163]. Recently, a gain of toxic function of mutant glucocerebrosidase was described, as it impairs alpha-synuclein degradation by blockade of chaperone-mediated autophagy [164]. Improving glucocerebrosidase-related lysosomal function was therefore suggested as disease-modifying strategy for PD, especially in patients that carry mutant *GBA1* alleles [134]. The small molecule isofagomine is chaperoning functional glucocerebrosidase to the lysosome where it releases the enzyme at low pH [37]. Administered orally for 4 months to Thy1-aSyn mice, isofagomine improved motor and non-motor function, abolished microglial inflammatory response in the substantia nigra, reduced alpha-synuclein immunoreactivity in nigral dopaminergic neurons, and shifted the distribution of alpha-synuclein aggregates from small to large [37]. Considering beneficial effects on behavioral readouts, small presynaptic alpha-synuclein aggregates may be toxic and impair neuronal function, whereas large aggregates in the soma of neurons could be innocuous or protective [37]. Increase of glucocerebrosidase expression via striatal AAV injection in Thy1-aSyn transgenics resulted in reduction of total alpha-synuclein and its aggregation and restored striatal dopamine transporter and tyrosine hydroxylase levels [165]. Behavioral effects were restricted to a subtle reduction of hyperactivity in the open field, but no effects on nest-building and olfaction [165].

Recently, NPT520-34, a small-molecule therapeutic candidate initially discovered in cell-based screening assays measuring the ability of compounds to reduce alpha-synuclein accumulation with an associated upregulation of LC3, a key protein involved in protein clearance mechanisms (proxy for autophagosomes), was tested in 3 months old male Thy1-aSyn mice [166]. Daily administration (i.p. daily Monday through Friday) of NPT520-34 increased LC3 abundance in the brain as expected (1 month treatment), reduced alpha-synuclein pathology, striatal denervation and Toll-like receptor 2 expression (3 months treatment), and improved gait abnormalities (1 month treatment) and grip strength (2.5 months treatment) [166]. Directly improving the protein degradation machinery is a very attractive target for PD, as it is likely to affect several detrimental pathways, and thus different etiologies, courses, and progression stages of disease, while less likely to produce unwanted side effects, especially if it is specific to brain lysosomes.

### Targeting Mitochondrial and Neurotransmitter Deficits in Thy1-aSyn Mice

Mitochondrial impairment and oxidative stress play central roles in the pathogenesis of PD. Treatment strategies involving various anti-oxidative agents showed exciting results in pre-clinical studies involving different animal models; however, clinical trial results were not convincing [167]. However, there are ongoing efforts using anti-oxidant, mitochondria-targeted, or metal-chelating compounds. For example, carnosine was tested in Thy1-aSyn mice to investigate effects on sensorimotor behaviors, transcriptome, and mitochondrial function [168, 169]. Carnosine is a dipeptide with anti-aggregating and metal-chelating properties. Intranasal or drinking water delivery ameliorated gene expression changes in the midbrain of Thy1-aSyn mice, with EIF2, eIF4, p70S6K, corticotropin-releasing hormone signaling, mTOR, and cAMP response element-binding protein (CREB) signaling most affected. On protein level, carnosine treatment promoted complex-IV respiration in Thy1-aSyn mice. In a follow up study, the authors further investigated safety of intranasal administration as well as behavioral outcomes of carnosine administration. Intranasal treatment did not alter olfactory function tested in the buried pellet test, but rescued motor impairment in the challenging beam traversal test [168, 169].

Cholesterol-oximes TRO19622 and TRO40303 target outer mitochondrial membrane proteins and were identified as potentially neuroprotective in cell-based assays of neurotrophic deprivation. Thy1-aSyn mice were fed with both compounds in food pellets for 3 months starting after weaning [99]. Unbiased weighted gene co-expression network analysis (WGCNA) of transcriptional changes in laser-captured dopamine neurons of the substantia nigra revealed effects of cholesterol-oximes on transcripts related to mitochondria, cytoprotection, and anti-oxidant response. High doses of TRO40303 improved olfaction but increased errors on the challenging beam test. Interestingly, the compound increased several transcripts involved in dopamine synthesis and reuptake, suggesting an increase in dopamine signaling, supported by the previous observation that dopamine enhancement in these young Thy1-aSyn mice specifically increases errors per step on the beam test. While gene expression results support neuroprotective properties of cholesterol-oximes, only TRO19622 resulted in an increase in the surface area occupied by alpha-synuclein aggregates [99].

Changes in the cholinergic system in PD provide an important target to treat motor and non-motor features [4]. Earlier studies discovered reduction of acetylcholine in cortex of Thy1-aSyn mice similar to PD patients [78, 170]. As cholinesterase inhibitors show limited efficacy in patients, stimulating cholinergic nicotinic receptors was evaluated as alternative approach [171]. Osmotic minipumps were used for steady long-term delivery (up to 5 months) of two concentrations of nicotine to Thy1-aSyn and wild-type mice. Nicotine administration improved cognitive and social performance of Thy1-aSyn mice in the Y-maze test, the novel object recognition test, and in the social approach task. Conversely, chronic nicotine treatment did not affect alpha-synuclein-induced motor deficits on the challenging beam and pole tests, or in the open field. Finally, chronic nicotine treatment did not affect alpha-synuclein pathology in Thy1-aSyn mice, nor striatal synaptic markers and microgliosis, which argues against a neuroprotective effect. The higher dose was already toxic in transgenic mice and could not be elevated further. In this study, repeated surgeries may have influenced endpoints, as there was less cognitive impairment in vehicle-treated transgenics and less expression of pathology markers as documented from other studies [171].

### Reducing Inflammation Markers in the Brain of Thy1-aSyn Mice

As described above, Thy1-aSyn mice replicate central inflammatory pathways thought to contribute to PD pathophysiology. Therefore, anti-inflammatory strategies are tested in this model. For example, cyclosporine A (calcineurin inhibitor) administration in Thy1-aSyn mice elicited improvement in motor and cognitive tasks, reduced human alpha-synuclein load and glial response, and partially restored the level of tyrosine hydroxylase in the striatum and increased levels of synaptic markers [172]. Interestingly, the authors discovered that cyclosporine A triggers autophagy and thereby promotes the clearance of alpha-synuclein [172]. Lenalidomide (thalidomide derivative) also improved pathology and behavioral endpoints in Thy1-aSyn mice [173]. Administration (oral gavage 5 times/week for 5 weeks starting at 9 months of age) of the immune-modulatory drug restored tyrosine immunoreactivity in the striatum of transgenic mice without affecting alpha-synuclein expression in striatum or hippocampus. Furthermore, lenalidomide reduced microglial Iba1 protein expression (but not astroglial Gfap) and pro-inflammatory TNFα, IL-6, IL-1β, and IFNγ mRNA expression, but increased expression of anti-inflammatory cytokine IL-10 mRNA among others, and reduced NF-κB p65 nucleus/cytosol ratio, indicating inhibition of NF-κB. Despite the lack of detectable effects on alpha-synuclein pathology, lenalidomide improved behavioral impairments observed in open field and the round beam test [173]. Given that microgliosis develops downstream of alpha-synuclein accumulation, beneficial effects on behavior may not necessarily require changes in aggregation. In reverse, this supports that microgliosis is critically involved in motor deficits in Thy1-aSyn mice. Previous studies showed that Toll-like receptor 2 (TLR2) activation results in inhibition of autophagy by regulation of the AKT/mTOR pathway, which reduced clearance of alpha-synuclein and promotes neurodegeneration [174]. Elevated TLR2 expression was found in pyramidal neurons of the neocortex as well as in astroglia and microglia cells in synucleinopathy patients and in Thy1-aSyn mice [175]. Anti-TLR2 antibody (T2.5) was injected intraperitoneally in 9 months old mice once a week for 4 weeks. Treatment reduced levels of IL-1β, TNFα, and IL-6 and NFκB activation in Thy1-aSyn transgenics. Furthermore, TLR2-antibody reduced alpha-synuclein pathology and exerted neuroprotection in the neocortex, hippocampus, and the striatum, and reduced the phenotype in the open field test [175].

## Final Remarks

PD is a complex syndrome and multiple neurodegenerative processes, which may spread across cells, drive its progression. Therefore, halting progression of PD via disease-modifying drugs is very challenging and requires further optimization of pre-clinical and clinical strategies (Fig. 1). With their large variety of endpoints in multiple behavioral domains (e.g., motor, cognition, anxiety, olfaction, gastrointestinal) and wide range of molecular disturbances similar to those known or suspected to occur in the majority of PD patients (e.g., dopamine loss, striatal degeneration, microgliosis, mitochondrial deficits, alpha-synuclein aggregation), the Thy1-aSyn line 61 model is a useful tool to test novel therapeutic strategies (Figs. 2 and 3). As any tool, its value is maximized by optimized utilization. Depending on the drugs’ mode of action, onset and duration of treatment and selection of most informative endpoints require an optimized design (Fig. 3). Interpretation of the functional readouts in conjunction with changes in pathology are as complex as the underlying brain circuitries and pathophysiological pathways. Therefore, extensive experience in a single model is of advantage, to compare across modes of action and pre-clinical trials, and to provide the best possible informed interpretation of observed effects. Of note, the better we understand how changes in pathophysiological pathways translate into behavioral and pathological readouts, the closer we get to reverse-engineered compound testing, i.e., predicting the target of an apparently neuroprotective drug from its pharmacodynamic responses in the model (Fig. 2).

Importantly, further optimization of the model has to be aligned with development of biomarkers and readouts in patients. There are no imaging markers for alpha-synuclein pathology in the brain, and for a given compound, it is not clear whether target engagement measured in cerebrospinal fluid or plasma replicates the conditions in the brain. To aid this interpretation, related studies in disease models should correlate brain, cerebrospinal fluid, and plasma end points. It appears imperative to assess and establish that a drug candidate can modify a specific biomarker in careful, dose-ranging animal studies. We recently tested venglustat in parallel to its clinical trial (MOVES-PD), with the same outcome in motor symptoms (worsening of motor deficits) together with increase in pathology specifically in the substantia nigra (manuscript in revision). While no model will encompass the entire complex PD syndrome, two decades of characterization of this model with its solid construct validity substantially increased our knowledge on underlying disease mechanisms. Thy1-aSyn line 61 now provides a useful platform for more informative pre-clinical studies.

## Supplementary Information

Below is the link to the electronic supplementary material.

Supplementary file1 (PDF 508 kb)

Supplementary file2 (PDF 516 kb)

Supplementary file3 (PDF 499 kb)

Supplementary file4 (PDF 534 kb)

Supplementary file5 (PDF 525 kb)

## Data Availability

The data that support the findings of this study are available from the corresponding author, FR, upon reasonable request.
